# Supplementary material for: Increased male investment in sperm competition results in reduced maintenance of gametes
Source: PLoS Biol. 2023 Apr 4;21(4):e3002049. doi: 10.1371/journal.pbio.3002049 (PMC10072457; doi:10.1371/journal.pbio.3002049)
Supplement: S1 Appendix — (DOCX) [file pbio.3002049.s001.docx]

**Supplementary Information for**

Increased male investment in sperm competition results in reduced maintenance of gametes

Mareike Koppik^1, 2^, Julian Baur^1^ & David Berger^1, *^

^1^Department of Ecology and Genetics, Animal Ecology, Uppsala University, Uppsala, Sweden

^2^Department of Zoology, Animal Ecology, Martin-Luther University Halle-Wittenberg, Halle (Saale), Germany

*Correspondence: david.berger@ebc.uu.se

**S1 Appendix: Sperm competition results**

Table of contents: Page

Figure S1: sperm competition results 2

Table S1a: P1 sperm defense: model specifications and results 3

Table S1b: P2 sperm offense: model specifications and results 4-5

Table S1c: P1+P2 total success: model specifications and results 6


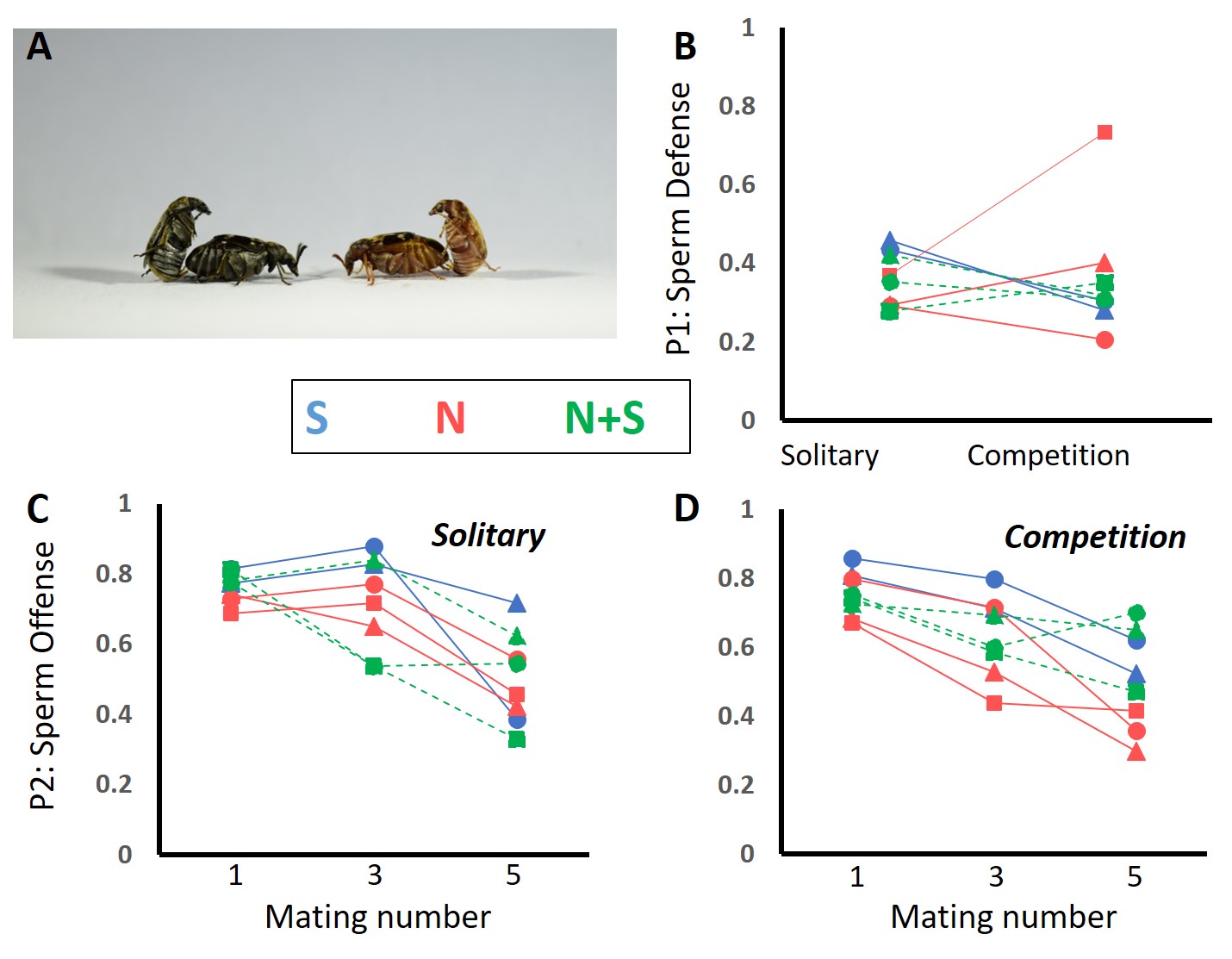


**Figure S1: Line specific sperm competition success in males from the experimental evolution lines.**

Shown are marginal means for each replicate line, averaged over experimental blocks. In A, paternity was estimated by competing a standard male deriving from a black strain (left) to focal males of each regime (right): S (blue), N (red) and N+S (hatched green). In B, paternity share when the focal male was first to mate (P1). In C and D, paternity share when the focal male was second to mate (P2). The data underlying this figure can be found in ‘SpermCompetition.txt’ at Mendeley Data (http://dx.doi.org/10.17632/szyb2z8rzk.1).

**Table S1a:** P1 – sperm defense**:** model specification and results

P1.mod.comp <- MCMCglmm(cbind(Wt,Black) ~ Regime,

random = ~ Line + Block,

data = P1.data[P1.data$Environment=="competition",],

family = "multinomial2", prior = priorP1sol, nitt=250000,

slice=TRUE, burnin=50000, thin=200, verbose = FALSE, pr=F)

DIC: 7222.24

G-structure: ~idh(Environment):Line

post.mean l-95% CI u-95% CI eff.samp

Environmentcompetition.Line 0.5885 0.0003493 2.5773 1000

Environmentsolitary.Line 0.0817 0.0002191 0.3705 1000

~Block

post.mean l-95% CI u-95% CI eff.samp

Block 0.2509 0.0002524 0.91 1000

R-structure: ~units

post.mean l-95% CI u-95% CI eff.samp

units 5.571 4.376 6.769 1725

Location effects: cbind(Wt, Black) ~ Regime * Environment

|  | post.mean | l-95%CI | u-95%CI | eff.samp | pMCMC |
| --- | --- | --- | --- | --- | --- |
| (Intercept)^a^ | -1.3917 | -2.8549 | 0.2601 | 827.2 | 0.086 |
| Regime.N | 1.1254 | -0.5683 | 3.3824 | 1287.4 | 0.222 |
| Regime.N+S | 0.2667 | -1.8107 | 2.1054 | 891.4 | 0.758 |
| Environment.solitary | 1.1752 | -0.5531 | 2.9016 | 820.9 | 0.158 |
| Regime.N:Environment.solitary | -2.0377 | -4.2689 | 0.1021 | 1000.0 | 0.052 |
| Regime.N+S:Environment.solitary | -1.1932 | -3.3884 | 1.2194 | 1000.0 | 0.248 |

^a^The intercept represents S-males after being held in competition (4 other males) 24 h prior to the sperm competition assay.

**Table S1b:** P2 – sperm offense**:** model specification and results

***Full model:***

P2.mod <- MCMCglmm(cbind(Wt,Black) ~ Regime*Environment + Regime*mating + Environment*mating,

#Note: Mating cannot be crossed with block because only M1 in B4-6

random = ~ idh(Environment):Line + mating:Line + ID + Block + Block:Environment,

data = P2.data, family = "multinomial2", prior = priorP2, nitt=2500000,

slice=TRUE, burnin=500000, thin=2000, verbose = FALSE, pr=F)

DIC: 20892.26

G-structure: ~idh(Environment):Line

post.mean l-95% CI u-95% CI eff.samp

Environmentcompetition.Line 0.10186 0.0002373 0.5000 708.8

Environmentsolitary.Line 0.09354 0.0004277 0.3824 1000.0

G-structure:

|  | post.mean | l-95%CI | u-95%CI | eff.samp |
| --- | --- | --- | --- | --- |
| mating:Line | 0.1741 | 0.0002315 | 0.5904 | 1000 |
| ID | 0.1605 | 0.0002638 | 0.6315 | 1000 |
| Block | 0.1237 | 0.0002298 | 0.5285 | 1000 |
| Block:Environment | 0.1826 | 0.0002924 | 0.5807 | 1000 |

R-structure: ~units

post.mean l-95% CI u-95% CI eff.samp

units 6.672 5.678 7.717 1000

Location effects: cbind(Wt, Black) ~ Regime * Environment + Regime * mating + Environment * mating

|  | post.mean | l-95% CI | u-95% CI | eff.samp | pMCMC |
| --- | --- | --- | --- | --- | --- |
| (Intercept)^a^ | 2.0630 | 1.0839 | 3.2500 | 1000.0 | 0.004 |
| Regime.N | -0.9029 | -2.3277 | 0.3207 | 1181.3 | 0.154 |
| Regime.N+S | -0.1766 | -1.4142 | 1.1270 | 1000.0 | 0.796 |
| Environment.solitary | 0.2404 | -0.8017 | 1.5715 | 1094.1 | 0.696 |
| mating3 | -0.2191 | -1.5969 | 1.2482 | 1687.8 | 0.736 |
| mating5 | -1.8972 | -3.3736 | -0.5436 | 1000.0 | 0.004 |
| Regime.N:Environment.solitary | 0.1971 | -1.0218 | 1.6660 | 1000.0 | 0.736 |
| Regime.N+S:Environment.solitary | -0.1722 | -1.6247 | 1.1331 | 1000.0 | 0.816 |
| Regime.N:mating3 | -0.5777 | -2.2911 | 0.9368 | 1095.3 | 0.450 |
| Regime.N+S:mating3 | -1.1803 | -2.7140 | 0.3937 | 1000.0 | 0.140 |
| Regime.N:mating5 | -0.1915 | -2.1180 | 1.2917 | 988.4 | 0.810 |
| Regime.N+S:mating5 | 0.3147 | -1.2141 | 2.0864 | 1000.0 | 0.670 |
| Environment.solitary:mating3 | 0.5334 | -0.3479 | 1.5704 | 1000.0 | 0.274 |
| Environment.solitary:mating5 | -0.2829 | -1.2422 | 0.8625 | 1114.1 | 0.614 |

^a^The intercept represents the first mating in S-males after being held in competition (4 other males) 24 h prior to the sperm competition assay.

***Final reduced model:***

P2.mod.ave <- MCMCglmm(cbind(Wt,Black) ~ Regime + Environment*mating,

#Note: Mating cannot be crossed with block because only M1 in B4-6

random = ~ idh(Environment):Line +

mating:Line +

ID + Block + Block:Environment,

data = P2.data,

family = "multinomial2", prior = priorP2, nitt=2500000,

slice=TRUE, burnin=500000, thin=2000, verbose = FALSE, pr=F)

DIC: 20893.94

G-structure: ~idh(Environment):Line

post.mean l-95% CI u-95% CI eff.samp

Environmentcompetition.Line 0.07729 0.0002482 0.3776 1000

Environmentsolitary.Line 0.06564 0.0002154 0.3017 1000

G-structure:

|  | post.mean | l-95% CI | u-95% CI | eff.samp |
| --- | --- | --- | --- | --- |
| mating:Line | 0.1492 | 0.0003136 | 0.485 | 1000 |
| ID | 0.1383 | 0.0003411 | 0.5294 | 1000 |
| Block | 0.1564 | 0.0003207 | 0.6505 | 1000 |
| Block:Environment | 0.1814 | 0.0002219 | 0.6066 | 1000 |

R-structure: ~units

post.mean l-95% CI u-95% CI eff.samp

units 6.638 5.652 7.59 1000

Location effects: cbind(Wt, Black) ~ Regime + Environment * mating

|  | post.mean | l-95%CI | u-95%CI | eff.samp | pMCMC |
| --- | --- | --- | --- | --- | --- |
| (Intercept)^a^ | 2.21063 | 1.32762 | 2.96763 | 808.9 | <0.001 |
| Regime.N | -0.96711 | -1.72442 | -0.21988 | 1000.0 | 0.012 |
| Regime.N+S | -0.49394 | -1.19405 | 0.25241 | 1000.0 | 0.192 |
| Environmentsolitary | 0.23958 | -0.48440 | 1.01106 | 1000.0 | 0.516 |
| matingM3 | -0.87431 | -1.76346 | -0.08077 | 1000.0 | 0.044 |
| matingM5 | -1.78452 | -2.71633 | -0.99419 | 1000.0 | <0.001 |
| Environmentsolitary:matingM3 | 0.52294 | -0.51514 | 1.47180 | 1000.0 | 0.314 |
| Environmentsolitary:matingM5 | -0.36921 | -1.31317 | 0.82827 | 871.6 | 0.506 |

^a^The intercept represents the first mating in S-males after being held in competition (4 other males) 24 h prior to the sperm competition assay.

**Table S1c**: Total success (P1 + P2): model specification and results

mod.P1P2 <- MCMCglmm(cbind(Wt,Black) ~ Regime + Paternity + mating + Environment ,

random = ~ Line + ID + Block + Block:Paternity + Block:Environment + Block:Paternity:Environment,

data = dat_all,

family = "multinomial2", prior = priorB, nitt=2500000,

slice=TRUE, burnin=500000, thin=2000, verbose = FALSE, pr=F)

DIC: 28121.69

G-structure:

|  | post.mean | l-95% CI | u-95% CI | eff.samp |
| --- | --- | --- | --- | --- |
| Line | 0.03634 | 0.0002584 | 0.1512 | 798.1 |
| ID | 0.1303 | 0.0002431 | 0.4965 | 1008 |
| Block | 0.1013 | 0.0002365 | 0.458 | 1000 |
| Block:Paternity | 0.0424 | 0.000282 | 0.1789 | 695.1 |
| Block:Environment | 0.1361 | 0.0002529 | 0.4249 | 1142 |
| Block:Paternity:Environment | 0.05179 | 0.0002102 | 0.212 | 723 |

R-structure: ~units

post.mean l-95% CI u-95% CI eff.samp

units 6.317 5.545 7.159 1000

Location effects: cbind(Wt, Black) ~ Regime + Paternity + mating + Environment

|  | post.mean | l-95% CI | u-95% CI | eff.samp | pMCMC |
| --- | --- | --- | --- | --- | --- |
| (Intercept)^a^ | -0.5793 | -1.2384 | 0.2094 | 1000 | 0.124 |
| Regime.N | **-0.7204** | **-1.2358** | **-0.1900** | **1000** | **0.012** |
| Regime.N+S | **-0.4657** | **-1.0167** | **0.1093** | **1000** | **0.088** |
| PaternityP2 | **2.6848** | **2.1595** | **3.2040** | **1000** | **<0.001** |
| matingM3 | **-0.6614** | **-1.1468** | **-0.1679** | **1000** | **0.008** |
| matingM5 | **-2.0185** | **-2.6009** | **-1.5051** | **1000** | **<0.001** |
| Environmentsolitary | 0.2066 | -0.3716 | 0.7946 | 1000 | 0.446 |

^a^The intercept represents sperm defense (P1) in the first mating in S-males after being held in competition (4 other males) 24 h prior to the sperm competition assay.
