# Supplementary material for: Increased male investment in sperm competition results in reduced maintenance of gametes
Source: PLoS Biol. 2023 Apr 4;21(4):e3002049. doi: 10.1371/journal.pbio.3002049 (PMC10072457; doi:10.1371/journal.pbio.3002049)
Supplement: S2 Appendix — (DOCX) [file pbio.3002049.s002.docx]

**Supplementary Information for**

Increased male investment in sperm competition results in reduced maintenance of gametes

Mareike Koppik^1, 2^, Julian Baur^1^ & David Berger^1, *^

^1^Department of Ecology and Genetics, Animal Ecology, Uppsala University, Uppsala, Sweden

^2^Department of Zoology, Animal Ecology, Martin-Luther University Halle-Wittenberg, Halle (Saale), Germany

*Correspondence: david.berger@ebc.uu.se

**S2 Appendix: Offspring quality**

Table of contents Page

Table S2a: Reduction in offspring quality after short-term recovery 2

Table S2b: Reduction in offspring quality after long-term recovery 3

**Table S2a: Reduction in offspring quality after short-term recovery period**

***Final reduced model***

Iterations = 200001:5195001

Thinning interval = 5000

Sample size = 1000

DIC: 3087.496

G-structure: ~mm(Dam_n + Sire_n)

post.mean l-95% CI u-95% CI eff.samp

Dam_n+Sire_n 0.0009809 1.56e-07 0.003903 384.8

R-structure: ~idh(Irradiation:Males:Mating):units

|  | post.mean | l-95% CI | u-95% CI | eff.samp |
| --- | --- | --- | --- | --- |
| ctrl:NoMales:Mated.units | 0.0006591 | 1.394e-07 | 0.003497 | 1000 |
| Irradiation:NoMales:Mated.units | 0.1381204 | 7.957e-02 | 0.201832 | 1000 |
| ctrl: OtherMales:Mated.units | 0.0102144 | 3.576e-07 | 0.020712 | 607 |
| Irradiation:OtherMales:Mated.units | 0.3371629 | 2.073e-01 | 0.502774 | 1000 |
| ctrl: NoMales:Virgin.units | 0.0114436 | 4.548e-07 | 0.023814 | 1000 |
| Irradiation:NoMales:Virgin.units | 0.2051549 | 1.248e-01 | 0.294872 | 1512 |
| ctrl: OtherMales:Virgin.units | 0.0201414 | 5.251e-03 | 0.034927 | 1000 |
| Irradiation:OtherMales:Virgin.units | 0.2757921 | 1.662e-01 | 0.397390 | 1000 |

Location effects: Offspring ~ Irradiation * Males + Irradiation * Mating + Irradiation * Line

|  | post.mean | l-95% CI | u-95% CI | eff.samp | pMCMC |
| --- | --- | --- | --- | --- | --- |
| (Intercept)^a^ | 4.643279 | 4.596021 | 4.687729 | 1000 | <0.001 |
| Irradiation | -0.591616 | -0.718332 | -0.475799 | 1105 | <0.001 |
| OtherMales | 0.001035 | -0.041375 | 0.051118 | 1000 | 0.988 |
| Virgin | -0.017207 | -0.069179 | 0.026461 | 1000 | 0.488 |
| Line.S3 | -0.005548 | -0.048280 | 0.042137 | 1000 | 0.794 |
| **Irradiation:OtherMales** | **-0.204506** | **-0.329832** | **-0.051711** | **1012** | **0.010** |
| **Irradiation:Virgin** | **0.121672** | **-0.023893** | **0.250948** | **1000** | **0.088** |
| Irradiation:Line.S3 | 0.218059 | 0.083391 | 0.363522 | 1000 | 0.002 |

^a^The intercept represents the control treatment with no other males but a female mating partner in the socio-sexual environment (group “mated”) in males from line S1.

**Table S2b: Reduction in offspring quality after long-term recovery period**

***Final reduced model***

Iterations = 200001:5195001

Thinning interval = 5000

Sample size = 1000

DIC: 9599.919

G-structure: ~mm(Dam_n + Sire_n)

post.mean l-95% CI u-95% CI eff.samp

Dam_n+Sire_n 0.00044 1.404e-07 0.002097 1000

R-structure: ~idh(Irradiation:Males:Mating):units

|  | post.mean | l-95% CI | u-95% CI | eff.samp |
| --- | --- | --- | --- | --- |
| ctrl: NoMales:Mated.units | 0.02293 | 0.01433 | 0.03216 | 1000.0 |
| Irradiation:NoMales:Mated.units | 0.60516 | 0.45373 | 0.75083 | 1000.0 |
| ctrl: OtherMales:Mated.units | 0.05947 | 0.03693 | 0.08438 | 1144.0 |
| Irradiation:OtherMales:Mated.units | 0.56492 | 0.42483 | 0.71519 | 1000.0 |
| ctrl: NoMales:Virgin.units | 0.06223 | 0.03912 | 0.08860 | 1000.0 |
| Irradiation:NoMales:Virgin.units | 0.55783 | 0.43234 | 0.70548 | 960.5 |
| ctrl: OtherMales:Virgin.units | 0.10257 | 0.06552 | 0.13963 | 1000.0 |
| Irradiation:OtherMales:Virgin.units | 0.50336 | 0.38070 | 0.63391 | 1000.0 |

Location effects: Offspring ~ Irradiation * Mating + Irradiation * Males + Irradiation * Line + Irradiation * Day

|  | post.mean | l-95% CI | u-95% CI | eff.samp | pMCMC |
| --- | --- | --- | --- | --- | --- |
| (Intercept) ^a^ | 4.6214789 | 4.5737013 | 4.6665965 | 1096.5 | <0.001 |
| Irradiation | -0.8551001 | -0.9992089 | -0.7289456 | 1124.5 | <0.001 |
| Virgin | -0.0358189 | -0.0849920 | 0.0084509 | 1000.0 | 0.130 |
| OtherMales | 0.0018353 | -0.0477967 | 0.0441239 | 964.0 | 0.930 |
| Line.S3 | 0.0290843 | -0.0119659 | 0.0720544 | 1000.0 | 0.180 |
| Day2 | 0.0010393 | -0.0468999 | 0.0451672 | 913.1 | 0.990 |
| **Irradiation:Virgin** | **0.1323434** | **0.0207648** | **0.2667268** | **1000.0** | **0.030** |
| **Irradiation:OtherMales** | **0.0116687** | **-0.1035681** | **0.1373192** | **1000.0** | **0.844** |
| Irradiation:Line.S3 | 0.1554541 | 0.0361867 | 0.2749441 | 1000.0 | 0.010 |
| Irradiation:Day2 | -0.1234450 | -0.2509863 | -0.0002705 | 1000.0 | 0.052 |

^a^The intercept represents the control treatment with no other males but a female mating partner in the socio-sexual environment (group “mated”) in males from line S1.
