# Supplementary material for: Increased male investment in sperm competition results in reduced maintenance of gametes
Source: PLoS Biol. 2023 Apr 4;21(4):e3002049. doi: 10.1371/journal.pbio.3002049 (PMC10072457; doi:10.1371/journal.pbio.3002049)
Supplement: S3 Appendix — (DOCX) [file pbio.3002049.s003.docx]

**Supplementary Information for**

Increased male investment in sperm competition results in reduced maintenance of gametes

Mareike Koppik^1, 2^, Julian Baur^1^ & David Berger^1, *^

^1^Department of Ecology and Genetics, Animal Ecology, Uppsala University, Uppsala, Sweden

^2^Department of Zoology, Animal Ecology, Martin-Luther University Halle-Wittenberg, Halle (Saale), Germany

*Correspondence: david.berger@ebc.uu.se

**S3 Appendix: Gene expression**

Table of contents Page

Figure S3: Venn diagram of main contrasts on gene expression analysis 2

Table S3a: Irradiation response 3

Table S3b: Sex bias in gene expression of irradiation responsive genes 4

Table S3c: Correlation of gene expression and sperm defense 5

Table S3d: Correlation of gene expression and sperm offense 6

Figure S3: Venn Diagram of differentially expressed genes (5 % false discovery rate) of the main contrasts (socio-sexual treatments are all compared to solitary males).

Table S3a: Test statistics and log_2_-fold change (irradiated - control) of the 18 genes being significantly differentially expressed between irradiated and control samples (at 5 % false discovery rate).

| **Gene** | **Log_2_-fold change** | **Average expression** | **t** | **P-Value** | **BH adjusted P-Value** | **B** |
| --- | --- | --- | --- | --- | --- | --- |
| *CALMAC_LOCUS19707* | -1.250 | 0.541 | -5.933 | 1.84E-06 | 0.003 | 4.840 |
| *CALMAC_LOCUS14* | -0.994 | 2.436 | -7.370 | 3.80E-08 | < 0.001 | 8.104 |
| *CALMAC_LOCUS18783* | 0.715 | 0.827 | 6.657 | 2.54E-07 | 0.001 | 5.280 |
| *CALMAC_LOCUS9667* | -0.711 | 3.069 | -5.804 | 2.63E-06 | 0.004 | 4.643 |
| *CALMAC_LOCUS15686* | -0.650 | 3.636 | -6.030 | 1.41E-06 | 0.003 | 5.288 |
| *CALMAC_LOCUS10093* | -0.502 | 5.119 | -5.522 | 5.76E-06 | 0.006 | 3.970 |
| *CALMAC_LOCUS17392* | 0.495 | 3.819 | 6.690 | 2.32E-07 | 0.001 | 6.980 |
| *CALMAC_LOCUS9612* | -0.456 | 2.424 | -4.796 | 4.37E-05 | 0.031 | 2.046 |
| *CALMAC_LOCUS9511* | 0.413 | 1.695 | 5.214 | 1.36E-05 | 0.012 | 2.862 |
| *CALMAC_LOCUS1251* | 0.352 | 6.058 | 8.944 | 7.12E-10 | < 0.001 | 12.569 |
| *CALMAC_LOCUS5314* | -0.338 | 2.601 | -5.572 | 5.01E-06 | 0.006 | 3.981 |
| *CALMAC_LOCUS8422* | -0.294 | 2.511 | -4.843 | 3.83E-05 | 0.029 | 2.171 |
| *CALMAC_LOCUS20262* | 0.248 | 3.280 | 5.246 | 1.24E-05 | 0.012 | 3.259 |
| *CALMAC_LOCUS10868* | 0.228 | 6.723 | 6.685 | 2.36E-07 | 0.001 | 7.029 |
| *CALMAC_LOCUS2860* | 0.227 | 4.565 | 4.871 | 3.54E-05 | 0.029 | 2.258 |
| *CALMAC_LOCUS10402* | 0.188 | 7.123 | 5.561 | 5.17E-06 | 0.006 | 4.062 |
| *CALMAC_LOCUS8201* | 0.185 | 5.119 | 5.422 | 7.61E-06 | 0.008 | 3.702 |
| *CALMAC_LOCUS1539* | 0.174 | 5.122 | 4.778 | 4.60E-05 | 0.031 | 1.980 |

Table S3b: Sex bias (male - female) in expression of the 18 irradiation responsive genes. Table represents a subset taken from the overall sex-bias analysis in all 12874 analyzed genes.

| **Gene** | **Log_2_-fold change** | **Average expression** | **t** | **P-Value** | **BH-adjusted P-Value** | **B** |
| --- | --- | --- | --- | --- | --- | --- |
| *CALMAC_LOCUS10093* | -0.700 | 6.141 | -11.883 | 1.34E-08 | 5.01E-08 | 9.298 |
| *CALMAC_LOCUS10402* | -0.906 | 7.672 | -15.586 | 4.13E-10 | 2.49E-09 | 12.887 |
| *CALMAC_LOCUS10868* | 0.467 | 6.849 | 8.823 | 5.06E-07 | 1.25E-06 | 5.373 |
| *CALMAC_LOCUS1251* | -0.549 | 6.519 | -10.298 | 7.89E-08 | 2.39E-07 | 7.376 |
| *CALMAC_LOCUS14* | -1.433 | 4.042 | -3.148 | 7.28E-03 | 9.73E-03 | -4.073 |
| *CALMAC_LOCUS1539* | -0.956 | 5.860 | -16.988 | 1.34E-10 | 9.85E-10 | 14.222 |
| *CALMAC_LOCUS15686* | 0.555 | 4.788 | 3.820 | 1.94E-03 | 2.77E-03 | -2.991 |
| *CALMAC_LOCUS17392* | -1.828 | 5.991 | -33.818 | 1.33E-14 | 9.54E-13 | 23.904 |
| *CALMAC_LOCUS18783* | -1.116 | 0.557 | -4.277 | 8.03E-04 | 1.19E-03 | -0.993 |
| *CALMAC_LOCUS19707* | 1.599 | -0.794 | 3.133 | 7.50E-03 | 1.00E-02 | -2.976 |
| *CALMAC_LOCUS20262* | -1.957 | 4.492 | -21.700 | 5.24E-12 | 7.33E-11 | 17.882 |
| *CALMAC_LOCUS2860* | -0.901 | 5.181 | -8.235 | 1.13E-06 | 2.60E-06 | 4.738 |
| *CALMAC_LOCUS5314* | -0.083 | 3.532 | -0.785 | 4.46E-01 | 4.75E-01 | -7.686 |
| *CALMAC_LOCUS8201* | -1.262 | 5.952 | -26.018 | 4.61E-13 | 1.17E-11 | 20.205 |
| *CALMAC_LOCUS8422* | -0.059 | 2.672 | -0.537 | 6.00E-01 | 6.26E-01 | -7.636 |
| *CALMAC_LOCUS9511* | -0.368 | 1.628 | -2.422 | 2.99E-02 | 3.72E-02 | -4.888 |
| *CALMAC_LOCUS9612* | 0.669 | 2.806 | 6.293 | 2.18E-05 | 3.98E-05 | 2.125 |
| *CALMAC_LOCUS9667* | 0.719 | 4.503 | 5.137 | 1.62E-04 | 2.61E-04 | -0.366 |

Table S3c: Pearson’s correlation coefficients and test statistics of individual correlation analyses between sperm defense success (P1) and expression of irradiation responsive genes in experimental evolution lines. Multiple testing correction of P-Values was done with Benjamini-Hochberg (BH) method.

| **Gene** | **Pearson's *r*** | **t_6_** | **P-Value** | **BH-adjusted P-Value** |
| --- | --- | --- | --- | --- |
| *CALMAC_LOCUS19707* | 0.08 | 0.209 | 0.842 | 0.947 |
| *CALMAC_LOCUS14* | 0.59 | 1.808 | 0.121 | 0.543 |
| *CALMAC_LOCUS18783* | -0.09 | -0.226 | 0.829 | 0.947 |
| *CALMAC_LOCUS9667* | -0.02 | -0.046 | 0.965 | 0.965 |
| *CALMAC_LOCUS15686* | -0.43 | -1.177 | 0.284 | 0.639 |
| *CALMAC_LOCUS10093* | -0.02 | -0.045 | 0.965 | 0.965 |
| *CALMAC_LOCUS17392* | 0.73 | 2.644 | 0.038 | 0.345 |
| *CALMAC_LOCUS9612* | -0.30 | -0.765 | 0.473 | 0.774 |
| *CALMAC_LOCUS9511* | -0.49 | -1.366 | 0.221 | 0.568 |
| *CALMAC_LOCUS1251* | -0.54 | -1.567 | 0.168 | 0.568 |
| *CALMAC_LOCUS5314* | -0.38 | -1.007 | 0.353 | 0.663 |
| *CALMAC_LOCUS8422* | 0.12 | 0.307 | 0.769 | 0.947 |
| *CALMAC_LOCUS20262* | 0.18 | 0.455 | 0.665 | 0.947 |
| *CALMAC_LOCUS10868* | 0.77 | 2.984 | 0.025 | 0.345 |
| *CALMAC_LOCUS2860* | -0.15 | -0.362 | 0.730 | 0.947 |
| *CALMAC_LOCUS10402* | -0.62 | -1.949 | 0.099 | 0.543 |
| *CALMAC_LOCUS8201* | 0.49 | 1.391 | 0.213 | 0.568 |
| *CALMAC_LOCUS1539* | -0.37 | -0.973 | 0.368 | 0.663 |

Table S3d: Pearson’s correlation coefficients and test statistics of individual correlation analyses between sperm offense success (P2) and expression of irradiation responsive genes in experimental evolution lines. Multiple testing correction of P-Values was done with Benjamini-Hochberg (BH) method.

| **Gene** | **Pearson's *r*** | **t_6_** | **P-Value** | **BH-adjusted P-Value** |
| --- | --- | --- | --- | --- |
| *CALMAC_LOCUS19707* | 0.16 | 0.398 | 0.704 | 0.906 |
| *CALMAC_LOCUS14* | -0.23 | -0.577 | 0.585 | 0.906 |
| *CALMAC_LOCUS18783* | -0.16 | -0.407 | 0.698 | 0.906 |
| *CALMAC_LOCUS9667* | -0.10 | -0.248 | 0.813 | 0.912 |
| *CALMAC_LOCUS15686* | -0.22 | -0.552 | 0.601 | 0.906 |
| ***CALMAC_LOCUS10093*** | **0.90** | **5.196** | **0.002** | **0.036** |
| *CALMAC_LOCUS17392* | 0.21 | 0.535 | 0.612 | 0.906 |
| *CALMAC_LOCUS9612* | 0.19 | 0.483 | 0.647 | 0.906 |
| *CALMAC_LOCUS9511* | 0.37 | 0.987 | 0.362 | 0.906 |
| *CALMAC_LOCUS1251* | 0.18 | 0.442 | 0.674 | 0.906 |
| *CALMAC_LOCUS5314* | -0.23 | -0.57 | 0.589 | 0.906 |
| *CALMAC_LOCUS8422* | 0.12 | 0.294 | 0.779 | 0.912 |
| *CALMAC_LOCUS20262* | -0.24 | -0.593 | 0.575 | 0.906 |
| *CALMAC_LOCUS10868* | 0.00 | 0.009 | 0.993 | 0.993 |
| *CALMAC_LOCUS2860* | 0.54 | 1.591 | 0.163 | 0.906 |
| *CALMAC_LOCUS10402* | -0.27 | -0.696 | 0.513 | 0.906 |
| *CALMAC_LOCUS8201* | 0.57 | 1.685 | 0.143 | 0.906 |
| *CALMAC_LOCUS1539* | -0.07 | -0.183 | 0.861 | 0.912 |
