# Supplementary material for: Increased male investment in sperm competition results in reduced maintenance of gametes
Source: PLoS Biol. 2023 Apr 4;21(4):e3002049. doi: 10.1371/journal.pbio.3002049 (PMC10072457; doi:10.1371/journal.pbio.3002049)
Supplement: Attachment — Submitted filename: REVISION_response to reviewers.docx [file pbio.3002049.s004.docx]

Dear Editors,

Thank you for the opportunity to revise and resubmit our manuscript entitled: “*Increased male investment in sperm competition results in reduced maintenance of gametes*” to *PLOS Biology*. We were happy to see the appreciation of our work by all three reviewers that provided many helpful comments for how to further improve our contribution. We have now attended to all these comments, and you will find our detailed point-by-point responses below, and a version of our manuscript where revisions are highlighted – we added a few clarifications and small edits of our own as well. Additionally, we also re-arranged our supplementary information to improve clarity.

Most importantly, we followed the advice of Reviewer#3 and re-analyzed our sperm competition data to report results not only for the total success in sperm competition (P1+P2), but now also separately for P1 and P2. We also provide more detail on how the social treatment and successive mating affected sperm competition success. This has resulted in a more detailed analysis that we hope will be interesting to researchers working on postcopulatory sexual selection. We note that the main conclusions are the same as before. We have edited the text on sperm competition results and the description of their statistical methods. We have also produced a new Figure 1 on the results, as well as a supplementary figure (available in S1 Appendix).

We feel very content with our current manuscript but should you have further suggestions for how to improve our contribution we would of course be willing to do so.

All authors have approved this resubmission. We look forward to your decision.

On behalf of all authors,

David Berger

Please note that as a condition of publication PLOS' data policy (<http://journals.plos.org/plosbiology/s/data-availability>) requires that you make available all data used to draw the conclusions arrived at in your manuscript. If you have not already done so, you must include any data used in your manuscript either in appropriate repositories, within the body of the manuscript, or as supporting information (N.B. this includes any numerical values that were used to generate graphs, histograms etc.). For an example see here: <http://www.plosbiology.org/article/info%3Adoi%2F10.1371%2Fjournal.pbio.1001908#s5>

We now added a sentence to each figure legend (Figure 1 and 3-6) directing readers to where this data can be found.

REVIEWS:

Reviewer #1:

In a multi-step experiment using experimental evolution lines (natural versus sexual selection), irradiation, and phenotypic and transcriptomic measurements, the authors found and confirmed a potential trade-off between male investments in sperm competition (via germline replication rate) and offspring quality (via germline mutation rate).

This is a great study that will be relevant to the field. If found the study to be rigorous and carefully conducted, and the paper reads well. I do not have any major criticism but suggest addressing a few minor issues:

Many thanks for these kind words and for your appreciation of our study!

L20     male bias (not hyphenated, though 'male-biased …' often would be)
done

L38     also here: sex differences
done

L66     I think this link to sperm competition success should be made more explicit. I agree that males may have a higher germline mutation rate than females, and it seems clear that higher germline replication rates can generate more sperm, which is indeed under selection through sperm competition. But these comparisons are either between sexes or (mostly) between species (with species that have higher levels of sperm competition producing more sperm and possibly at a higher rate). Translating such arguments to variation within a natural population of a single species (where sperm competition occurs and offspring quality is under selection), however, seems a stretch to me. Is there any good evidence to suggest that some males replicate their germline at a substantially higher rate than others of the same population? Variation in mutation rates between males I can see, but more often, I would argue, differential sperm production stems from variation in testes size (accommodating
fewer or more germlines in parallel) but not necessarily from different rates of cell division. Can you please clarify?

We agree with the reviewer that it is unknown how much variation there is in replication rates per germ cell within species (for an individual of a given age). There is certainly variation in sperm maturation rates, as for example demonstrated by systematic differences between our evolution regimes used here (see: Baur & Berger 2020, Nature Ecol Evol). We can however not know whether those differences are underlain by differences in the number of replicating germlines or by the rate of replication, as the reviewer points out. In any case, in both these cases we expect trade-offs with maintenance, and in both cases the trade-off may well include both soma and germline.

We did not mean to imply that we expect the rate of germline replication to be the main driver of mutation rate within species. Rather, we intended to say that this has been a main hypothesis for variation in mutation rate between species, but that this is a rather simplified view and that maintenance process are at least as important in dictating the mutation rate. We then argue that such maintenance process, and trade-offs with producing more sperm and/or other ejaculate components conferring and advantage in sperm competition, are likely drivers of variation in mutation rate both within and between species. We have made some edits to this paragraph to clarify this (lines 72-77 in annotated version).

L69     division -> divisions
done

L156    How did you control for mating activity in the treatments with mating partners? This would be good to mention here as the potential to mate and actually copulating is not the same here and could influence the interpretation of the results.

Thanks for pointing this out. Within the groups that also contained competitors, the number of matings each male achieved might indeed be different. We had discussed this point during the design of the experiment. As it was not feasible to control the number of matings during the 24 h interaction phase, we settled on using all males that competed for the same set of females (though they were not strictly independent from each other), to get an average response of males that had none to a few prior matings. As the male to female ratio was kept constant, we expect this approach to mostly affect the variance rather than the mean of our measurements. Note also that females are eager to mate when virgin, and a mating in C. maculatus takes several minutes. Therefore, with the scramble competition mating system that this species exhibits, most males are likely to have mated at least once in this treatment, although we expect a few males (over the entire sample) to still be virgin follow the 24h. We have added a clarifying sentence to this section.

L171    In Bayesian statistics, the error bar around posterior means is typically referred to as 95% credible interval, not 95% confidence interval
corrected

L183    found instead of 'find' for consistency in tense
done

L186    One of the key points of Bayesian statistics is to move away from P-values and significance thresholds, but instead focus more on the 'degree of belief' in an event. Reporting P-values and interpreting them as 'marginally non-significant' thus seems to be at odds with the Bayesian framework.
We have made substantial edits to the part on Sperm competition and its associated statistics. We now refrain from using terminology such as “marginally (non)significant”. We note, however, that we used very weak and non-informative priors. Within this realm, the actual difference between results from Bayesian and ML approaches are very similar (ideally identical) and the differences remain more philosophical. The real reason for using Bayesian mixed models in our case was that the MCMCglmm package is superior in dealing with random terms with low number of levels (evolution lines in our cases) and deals better with overdispersion than many corresponding ML alternatives (which produce highly anticonservative estimates of statistical significance).

L207    DEG should be defined at first mention - it is widely known, but not everyone is familiar with transcriptomics terminology
done

L368    damage response is not hyphenated
done

L380    in -> is (before known)
done

L395/411        space missing before citation
done

L564    (and throughout) Petri dish is usually capitalized (it's a name)
changed everywhere

L578f   It might be helpful to briefly explain Middle-Class Neighbourhood breeding design here.
We have added a short explanation for how the MCN works (lines 919-920) and now also refer the readers to more detailed information in the publication listed below.

Shabalina, Svetlana A., Lev Yu Yampolsky, och Alexey S. Kondrashov. ”Rapid Decline of Fitness in Panmictic Populations of Drosophila Melanogaster Maintained under Relaxed Natural Selection”. *Proceedings of the National Academy of Sciences* 94, nr 24 (25 november 1997): 13034–39. <http://www.pnas.org/content/94/24/13034>.

We have also added a short explanation for the sex-specific MCN (lines 741-746 in annotated version) and refer the readers to more detailed information in the publications listed below.

Martinossi‐Allibert, Ivain, Emma Thilliez, Göran Arnqvist, och David Berger. ”Sexual Selection, Environmental Robustness, and Evolutionary Demography of Maladapted Populations: A Test Using Experimental Evolution in Seed Beetles”. *Evolutionary Applications* 2019. <https://doi.org/10.1111/eva.12758>.

Morrow, E. H., A. D. Stewart, och W. R. Rice. ”Assessing the Extent of Genome-Wide Intralocus Sexual Conflict via Experimentally Enforced Gender-Limited Selection”. *Journal of Evolutionary Biology* 21: 1046–54. <https://doi.org/10.1111/j.1420-9101.2008.01542.x>.

Reviewer #2:

This is an outstanding study that will surely impact the field in a very positive way, and that provides a leap in our understanding of reproductive trade-offs, male-female coevolution and sexual selection at large. In this paper, the authors combine a set of behavioural and fitness assays using beetles from experimentally evolved lines with gene-expression studies and tests of germline damage and maintenance. The main aim is to provide causal evidence that sperm competition may favour increased male germline replication rates, but at the cost of greater germline mutation rates and thus germline maintenance and repair. Their dataset provides solid and consistent evidence that this is the case, all the more impressive because results from different empirical approaches combine and complement each other beautifully. All in all, this study provides tantalizing evidence in favour of an idea that could potentially explain the widespread sex-bias in germline mutation
rates observed across the tree of life. In this sense, this study will very likely open up a novel avenue of research. This is the type of study you review and immediately gets you thinking about interpretation of your own results and implications for future work. The design of experiments is sound, the analyses are well-grounded, and the manuscript is very well written, with a very nice introduction and a nuanced and balanced discussion. As a cautionary note, I am not an expert on gene-expression studies, so I can't provide informed feedback on the methods used to this regard. Below I list a series of minor suggestions with the aim of improving an already excellent piece of work. My sincere congratulations to the authors.

Thanks your for the encouragement and helpful comments!

Line 40. I suggest re-writing this part. "The choice of an individual" is ambiguous and can lead to misunderstandings that can be easily avoided.

We have replaced “choices” with “allocation decisions”, which we hope should make it clear that we are taking about evolutionary optimization and not “conscious” decision making.

Line 123. The authors should briefly discuss what the consequences of having just two replicate EE lines may have for the interpretation of their results. Three EE replicates is already on the low side of EE replication and thus it seems some justification here is warranted.
We now address this issue when mentioning the number of replicate lines (lines 134-139 in annotated version).

Line 167. Here and in the legend for figure 3 the use of "virgin" is awkward and misleading. These males were only virgin during one part of the assays (obviously, as virgin males cannot have offspring). I suggest re-wording to "solitary" or "isolated".
We agree, we therefore changed all instances of virgin to solitary.

Lines 360-378. I miss here a sentence about how upregulated genes in females overlap with up/downregulated genes in response to radiation. This is a very nice result and should be highlighted here too. It also fits very nicely with the overarching idea of the study.

Thanks for the suggestion, we now added this result to the second paragraph of the discussion.

Line 380. "is" instead of "in" before "known".
done

Line 396. Cite Hopkins et al. 2019 PNAS here too, it is a very relevant paper that also backs up what you're stating here ("Divergent allocation of sperm and the seminal
proteome along a competition gradient in Drosophila melanogaster").
thanks for bringing this to our attention, we added the relevant citation (line 625 in annotated version)

Line 466. Perhaps delete the comma after "both"?
done

Line 482. Explain or provide a suitable reference.
We have added a short explanation for a sex-specific MCN works and now also refer the readers to more detailed information in the publications listed below (lines 741-746 in annotated version).

Martinossi‐Allibert, Ivain, Emma Thilliez, Göran Arnqvist, och David Berger. ”Sexual Selection, Environmental Robustness, and Evolutionary Demography of Maladapted Populations: A Test Using Experimental Evolution in Seed Beetles”. *Evolutionary Applications* 2019. <https://doi.org/10.1111/eva.12758>.

Morrow, E. H., A. D. Stewart, och W. R. Rice. ”Assessing the Extent of Genome-Wide Intralocus Sexual Conflict via Experimentally Enforced Gender-Limited Selection”. *Journal of Evolutionary Biology* 21: 1046–54. <https://doi.org/10.1111/j.1420-9101.2008.01542.x>.

Line 491. This seems to imply females mated three times overall. Please re-write to avoid confusion.
We see how this can cause confusion and rewrote this sentence.

Line 501. You mean emerged beetles here, but the wording implies you're freezing beans.
We do freeze the beetles with the beans, but the reviewer is correct in assuming the aim is to freeze the emerged beetles, we thus rewrote this sentence.

Line 504. Clarify by explicitly stating that pairs were separated after the end of matings.
done

Line 512. Explicitly state that males mated with different females, so that each female only mated twice. This is implied but it needs to be unambiguously stated to avoid misunderstandings (if males mated repeatedly with the same female this would conflate other effects).
done

Line 596. Spell out HPD.

done

Reviewer #3:

Overall I thought this was an interesting paper. In general it is well written and the results of broad significance
I have a few comments that are quite minor - but nonetheless quite important. My main concern relates to the level of detail around the sperm competition assays (and how they were analysed). The reader needs to know (have confidence) that there was some control over the age and mating status of the focal males, the competitor males and the females. This is important as male age and mating status can affect ejaculatory traits in this species.

Thanks for highlighting missing points in our Methods section, we now expanded this section to address the mentioned concerns. Age and mating status of all beetles used for the sperm competition experiment was kept the same (virgin individuals, age randomly varying for a maximum of 24 h for focal males and 48 h for all other beetles used in the experiment).

How the data were analysed was somewhat confusing to me. It does all look rather complicated, for what is fundamentally a quite simple study. It is not clear to me how the model (S2) actually works. I am unsure how a model of 'sperm competition success' can have a fixed effect of number of matings, when this applies (as far as I can tell) to the sperm offense part of 'sperm competition success'? Why not analyse sperm defense and sperm offense separately?

We agree that going into detail will be interesting for many people here. Therefore, we have analyzed and report P1 and P2 separately, which is now the main bulk of the results on sperm competition (Lines 195-222 in annotated version, Figure 1). We have updated both Results and Figure 1 as well as the description of statistical methods (Lines 823-844 in annotated version). We still report the results of the global model (P1+P2). In the global model, we blocked out the main effect of mating number, which was only assayed for P2. It is thus incorporated as a main effect which is possible to account for in this way (note that we assayed the first mating for both P1 and P2). The results are now more detailed, but the main conclusions remain the same.

Line 133: I think it is misleading to suggest a result in which p=0.078 is a strong tendency for higher success. I have similar issues with the marginal approach of a p = 0.088 on lines 184 to 186. I appreciate you use the caveat marginally non-significant, but the essence of meaning can be promoted via the use of terms such as strong tendency or by actually saying there was a decrease in offspring quality. Mating did not decrease offspring quality after the 3h recovery period - your study failed to reject the null-hypothesis. I would prefer a more conservative approach when dealing with violations of statistical orthodoxy.

We have made substantial edits to the part on Sperm competition and its associated statistics in response to your previous comment. We agree with your comments and now refrain from using terminology such as “marginally (non)significant” and instead say that effects are not significant and sometimes point out that there is a strong tendency when warranted (note that the low number of replicate lines limits statistical power, so sometimes we do feel that patterns are worth mentioning despite not being significant, but we try to be careful with our wording.

Line 135: p-value? By providing p-values the reader can decide on marginal v non-marginal calls.

See above

line 136: p-value?

See above

Line 137: Did prior matings affect success in sperm competition?

Yes. Now clarified (it lowers P2: L 220-222 in annotated version, Fig 1).

Line 137: " Similarly, the sperm competition advantage of S males was not significantly different for offense and defense" -what exactly does 'sperm competition advantage' mean? The P1 and P2 values are very different - how do I get the ns result from looking at the table in S2? It is not very clear

This referred to the relative difference between the S-regime and the other regimes for P1 and P2, respectively. However, we have now changed the presentation of the results so this does no longer apply. We have explained that all pairwise comparisons are calculated by extracting marginal means from the posterior distribution of the models. P-values may therefore not be directly visible in model summaries as these present model coefficients (which is the standard in MCMCglmm). We have added a more detailed explanation to the statistical methods (lines 838-844 in annotated version).

line 162: Figure 2 - it seems unusual to have fig 2A, whch presents data from Baur & Berger 2020?

While we generally agree with this comment, those previous results heavily motivated the approach of this study (see also response to your last point). Hence, we decided to include the figure rather than expecting readers to look up the reference. We would therefore prefer to keep the figure in the manuscript.

Fig 2 is also a little misleading in that it looks as if the quality of F1 offspring (and not F2) are being assayed for quality.

The assay itself is in fact capturing the quality of both F1 (their fecundity and fertility) and F2 (their juvenile to adult survivorship). We have clarified this in the figure legend.

line 250: 'revealed differences between social environments' - did it? They look remarkably similar in fig 4C. Based on what statistical evidence?

If you look at that figure you do indeed see consistent differences between sociosexual treatments. However, we note that it was a graphical illustration of the pattern, and not a formal statistical test for treatment differences, which was provided just before this section in the MANOVA based on the 18 candidate genes. We have re-written this sentence to make things clearer. The sentence now reads:

*“This graphical separation of irradiated from control samples recapitulated the statistical differences found between the social environments in the MANOVA.”*

line 362: could this be some type of paternal effect via nutrients transferred within the ejaculate? Being in a socio-sexual environment for 24h (quite a long time for an insect that lives for about a week or so as an adult) will use up resources, which will detract from investment in the ejaculate (reduced size, reduced nutrients, reduced water content) - all of which females may use to invest in offspring

Our hypothesis of course assumes that our observations are a result of male allocation decisions in response to its current or future social environments, some of which may be changes in ejaculate composition that affect the ability of the germline to repair damage. However, all males from all regimes (both control and irradiated) were exposed to the socio-sexual treatment. Thus, a direct effect of nutrients cannot explain the observed variation in offspring quality (estimated as the difference between lineages deriving from control and irradiated grandfathers) across sociosexual treatments. Interesting to note in this aspect is also that female fertility under benign conditions (as used here) is very little affected by male mating number (causing male ejaculate depletion) in *C. maculatus* (see e.g. Rönn et al. 2008).

In addition to this, the previous study by Baur and Berger (Fig. 2) showed that the reduction in germline maintenance (measured by assaying irradiated lineages) can only be seen in the S regime. If the response was a simple physiological phenomenon stemming from depleted ejaculate resources, we would have expected males from all lines to at least show some degree of the same effect (especially since their investment in overall ejaculate volume over repeated matings is similar; see Baur & Berger 2020). By including the gene expression data, we could show that expression changes in genes with associations to germline repair in response to the damage induced by irradiation differed between socio-sexual contexts and predicted changes in offspring quality. In our view, this lends further credibility to the hypothesis that germline maintenance plays a key role in the observed decrease in offspring quality.

Baur, Julian, och David Berger. ”Experimental Evidence for Effects of Sexual Selection on Condition-Dependent Mutation Rates”. *Nature Ecology & Evolution* 4, nr 5 (maj 2020): 737–44. <https://doi.org/10.1038/s41559-020-1140-7>.

Rönn, J. L., M. Katvala, och G. Arnqvist. ”Interspecific Variation in Ejaculate Allocation and Associated Effects on Female Fitness in Seed Beetles”. *Journal of Evolutionary Biology* 21, nr 2 (2008): 461–70. <https://doi.org/10.1111/j.1420-9101.2007.01493.x>.
